# Recent life events pose greatest risk for onset of major depressive disorder during mid-life

**DOI:** 10.1016/j.jad.2011.10.041

**Published:** 2012-02

**Authors:** Bauke T. Stegenga, Irwin Nazareth, Diederick E. Grobbee, Francisco Torres-González, Igor Švab, Heidi-Ingrid Maaroos, Miguel Xavier, Sandra Saldivia, Christian Bottomley, Michael King, Mirjam I. Geerlings

**Affiliations:** a Julius Center for Health Sciences and Primary Care, University Medical Center Utrecht, the Netherlands; b Medical Research Council General Practice Research Framework, UK; c Research Department of Primary Care and Population Health, UCL, UK; d Centro de Investigación Biomedica en Red de Salud Mental (CIBERSAM), Departmental Section of Psychiatry and Psychological Medicine, University of Granada, Spain; e Department of Family Medicine, University of Ljubljana, Slovenia; f Faculty of Medicine, University of Tartu, Estonia; g Faculdade Ciências Médicas, University of Lisbon, Portugal; h Departamento de Psiquiatra'ıa y Salud Mental, Universidad de Concepción, Chile; i Infectious Diseases Epidemiology Unit, LSHTM, UK; j Research Department of Mental Health Sciences, UCL, UK

**Keywords:** Major depression, Age, Stress, Interaction, Cohort

## Abstract

**Background:**

The authors examined an additive model for the association of life events and age with onset of major depressive disorder (MDD) and whether the combination of life events and age posed greater risk than the sum of their independent effects.

**Methods:**

Data were used from a prospective cohort study of 10,045 general practice attendees (PredictD). We included those without MDD at baseline (N = 8293). We examined age divided into tertiles and into 10 year groups. Life events were assessed at baseline using the List of Threatening Life Experiences Questionnaire and categorized according to type. Main outcome measure was onset of DSM-IV MDD at 6 or 12 months of follow-up. The authors calculated Relative Excess Risks due to Interaction (RERI).

**Results:**

6910 persons (83.3%) had a complete follow-up, of whom 589 (8.5%) had an onset of MDD (166 younger, 254 middle aged and 169 older). The combined effect of personal problems (RERI = 1.30; 95% CI 0.29 to 2.32), events in family or friends (RERI = 1.23; 95% CI 0.28 to 2.19), or problems with law (RERI = 1.57; 95% CI 0.33 to 2.82) and middle age was larger than the sum of individual effects.

**Limitations:**

Lower response to recruitment in the UK and the Netherlands.

**Conclusions:**

Recent life events carry the largest risk of onset of MDD in mid-life. Understanding the different vulnerability to life events according to age may help to indicate groups at a particular risk and assist in preventive strategies.

## 1 Introduction

Major depressive disorder (MDD) is a serious health problem and will be the second leading cause of burden of disease worldwide by 2030 (Mathers and Loncar, 2006). MDD has severe personal and public health consequences. To be able to prevent MDD, insight in risk factors for the onset of MDD is of clear importance. A body of research has shown that major life events may lead to onset of MDD (Paykel, 2001). It has also been suggested that the interaction between vulnerability factors and life events plays a role in the onset of MDD. For example, one study showed that vulnerability factors such as lack of employment and early loss of mother largely influenced whether or not a life event resulted in depression (Brown and Harris, 1978). Another study reported that women were approximately three times more likely to become depressed than men when a life event occurred (Maciejewski et al., 2001). These findings suggest that some people may be more vulnerable to onset of MDD than others, which is in accordance with the vulnerability-stress model (Holmes and Rahe, 1967; Krantz et al., 1985; Masuda and Holmes, 1967; Ormel, 1999). In general, this model suggests that vulnerability and stress factors interact to cause the disorder. For instance, with higher a priori vulnerability, lower levels of stress may be needed to become depressed. Few studies have examined whether there is interaction of life events with age in the onset of MDD, although the frequency of life events and vulnerability to depression may differ throughout life (Jorm, 2000; Kessing et al., 2003; Kessler et al., 2010a, 2010b; King et al., 2008a). Studies that have examined interaction between life events and age found inconsistent results. One study reported that maternal loss had a greater impact on the risk of onset of depression in those who were younger compared to those who were older, while another study showed that recent life events may have the strongest effect on depression in mid-life (Jordanova et al., 2007; Surtees and Wainwright, 2000). Two other studies did not find an interaction between age and life events on the risk of depression (Kessing et al., 2003; Oei and Zwart, 1990). These studies were limited by a small number of patients, a cross-sectional or retrospective design, or a narrow age range (Jordanova et al., 2007; Kessing et al., 2003; Oei and Zwart, 1990; Surtees and Wainwright, 2000). To examine the interaction between age and life events on the risk of onset of depression, large prospective studies with a reliable registration of life events and a wide age range are needed (Kessing et al., 2003). Our aim was to examine the association of recent life events and age with onset of major depressive disorder and whether the combination of life events and age posed greater risk than the independent effects of life events and age.

## 2 Material and methods

### 2.1 Study setting and design

PredictD is a multicenter prospective cohort study from which a multifactor algorithm was developed to predict risk of onset of major depressive disorder in 6 European countries and Chile, and has been described in greater detail elsewhere (Bottomley et al., 2010; King et al., 2006, 2008a, 2008b, 2011; Stegenga et al., 2010, 2011). The study was conducted in seven countries: 1) 25 general practices in the Medical Research Council's General Practice Research Framework, in the United Kingdom; 2) nine large primary care centers in Andalucía, Spain; 3) 74 general practices nationwide in Slovenia; 4) 23 general practices nationwide in Estonia; 5) seven large general practice centers near Utrecht, the Netherlands; 6) two large primary care centers, one in the Lisbon area (urban) and the other in Alentejo (rural), Portugal; and 7) 78 general practices in Concepción and Talcahuano in the Eighth region of Chile. The study was approved by local ethical committees in each participating country.

### 2.2 Study participants

Consecutive attendees aged 18 to 75 years were recruited (N = 10,045) and interviewed between April 2003 and September 2004 in Europe and between October 2003 and February 2005 in Chile, and re-interviewed after 6 and 12 months. Exclusion criteria were an inability to understand one of the main languages involved, psychosis, dementia and incapacitating physical illness. Recruitment differed slightly in each country because of local service preferences. In the UK and the Netherlands, researchers approached patients waiting for consultations, whereas in the other countries doctors first introduced the study before contact with the research team. All patients gave written informed consent.

### 2.3 Outcome measure

A diagnosis of major depressive disorder (MDD) in the preceding 6 months was assessed at baseline, 6 and 12 months in all patients according to DSM-IV criteria using the depression section of the Composite International Diagnostic Interview (CIDI) (American Psychiatric Association, 1994; WHO, 1997).

### 2.4 Life events

Major life events in the preceding 6 months were assessed at baseline using the self-report List of Threatening Life Experiences Questionnaire (Brugha et al., 1985). First, we examined the number of life events and categorized them into 0, 1 and 2 or more life events. Second, we extended the work by Brugha et al. by categorizing the 12 life events into 5 groups according to type of life event. Each life event group was then dichotomized into presence or absence of the life event:

1) Personal problems (suffered a serious illness, assault or injury);
2) Relational problems (separated due to marital difficulties, broke off a steady relationship, or a serious problem with a close friend, neighbor or relative);
3) Work related and financial problems (unemployed or seeking work unsuccessfully for more than 1 month, sacked from your job, or a major financial crisis);
4) Severe events in family or friends (a serious illness, assault, injury to or death of a parent, child, partner, close family friend or another relative);
5) Problems with law (problems with the police or court appearance, or something valuable was lost or stolen).

### 2.5 Other variables

Age, sex, level of education and country were assessed using self-report questionnaires at baseline. Higher education was defined as secondary school or higher, while lower education included primary school, trade or no education.

### 2.6 Data analysis

Of 10,045 participants at baseline, 395 persons were dropped from the analysis because they were younger than 18 years or older than 75 years or had missing data on age or CIDI diagnosis. We included participants who had no MDD in the 6 months prior to baseline (N = 8293). Onset was defined as a diagnosis of MDD between baseline and 6 months or between 6 and 12 months of follow-up.

Baseline characteristics for the sample with no MDD at baseline were calculated as means with standard deviations (SD) for continuous variables and numbers with percentages for categorical variables. We divided age in tertiles: younger, middle aged or older and calculated baseline characteristics according to these age groups. We also divided age into 10 year groups based on our previous work (King et al., 2011). Characteristics were also calculated for those with and without onset of MDD at 6 or 12 months of follow-up. In addition, we used logistic regression models in which onset of MDD was the dependent variable to calculate Odds Ratio's (OR) with accompanying confidence intervals (CI) for the following variables at baseline: age, sex, level of education, country, number of life events and categories of life events.

In the present analyses we were interested in examining whether the effect of life events on the risk of onset of MDD was different for different age groups, i.e. whether there was an interaction between age (vulnerability factor) and life events (stressor). Most often, interaction is assessed by the addition of a product term in a statistical model. In logistic regression analysis the coefficient associated with this product term quantifies the departure from multiplicativity (Knol et al., 2007). We were interested in identifying interactions on an additive rather than multiplicative scale as it has been argued that biological interactions can best be estimated by departure from additivity and this better reflects the vulnerability-stress model (Darroch, 1997; Kalilani and Atashili, 2006; Rothman and Greenland, 1998). To measure the amount of interaction on an additive scale we calculated the Relative Excess Risk due to Interaction (RERI) and accompanying confidence intervals (CI) using the delta method (Hosmer and Lemeshow, 1992; Kalilani and Atashili, 2006; Rothman and Greenland, 1998). If the CI does not include 0, the RERI is statistically significant and thus departure from additivity is present, i.e. the combined effect of age and life events is larger than the sum of age and life events separately.

We used logistic regression models to obtain both crude and adjusted estimates of the RERI. In these models onset of MDD at 6 or 12 months of follow-up was the dependent variable, and the following indicators (dummy variables) were included as independent variables: 1) no life event and young age (reference) [A−B −]; 2) no life event and middle age [A−B +]; 3) presence of a life event and young age [A+B −]; and 4) presence of a life event and middle age [A+B +]. We calculated RERIs using the following formula:

$$\text{RERI}=OR_{\text{A+B +}}-OR_{\text{A+B-}}-OR_{\text{A-B +}}+1$$

where OR_A+B +_, OR_A+B −_, OR_A−B +_ are odds ratios obtained from the logistic regression comparing groups A+B+, A+B − and A−B + with the reference group. We also calculated the RERI for the combination of life event and older age, where a dummy variable was created in a similar way as described above with the same reference group of absence of life events and young age. The RERI for middle age and the RERI for older age are based on separate analyses for better interpretation. Adjusted estimates of RERI were obtained by including a priori confounders sex, level of education and country in the models and using the resulting adjusted odds ratios in the formula given above. Our previous work has shown that there was negligible clustering (intraclass correlation = 0.003) within GP practices (King et al., 2011). Therefore we did not adjust our analyses for practice-level clustering. Confidence intervals for RERI were obtained using the delta method (Hosmer and Lemeshow, 1992). We also calculated the RERI for all life event groups and each 10 year age group. To examine whether the results were different in women than in men, we stratified our results by sex (i.e. analyzing women and men separately). We also repeated the analyses in those without a lifetime history of depressive symptoms to measure the amount of interaction on the risk of a possible first onset of MDD at 6 or 12 months of follow-up. A lifetime history of depressive symptoms was ruled out if the screen for two core symptoms of the lifetime CIDI depression section were absent. If one or two of the core symptoms were present, participants were considered to have a lifetime history of depressive symptoms. We used the same methods as mentioned above to calculate the crude and adjusted RERIs. All analyses were complete-case analysis, because missing data on covariates were few. Analyses were performed using PASW version 17 (IBM SPSS Statistics).

## 3 Results

Baseline characteristics for the study population (N = 8293) according to age group are presented in Table 1. Compared to the young age group (aged 18 to 40 years), persons in the middle age (aged 41 to 57 years) and older age (aged 58 to 75 years) groups were less likely to have experienced two or more life events. They were more likely to have experienced personal problems, but less likely to have experienced work related or financial problems, severe events in family or friends, or problems with law.

**Fig. 1:**
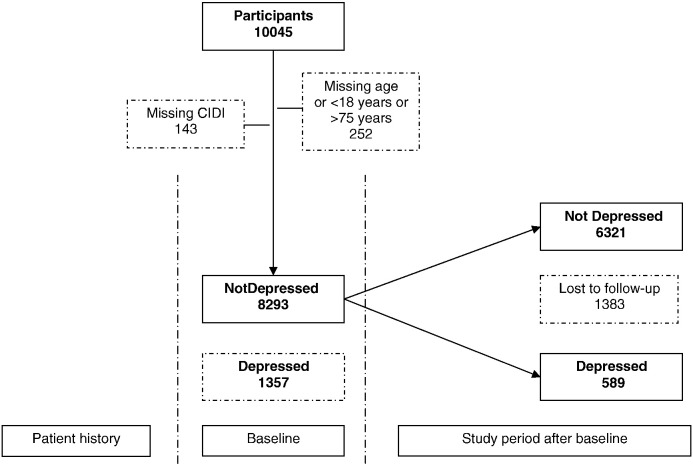
Flowchart of participants without major depressive disorder (MDD) in the 6 months prior to baseline who have an onset of MDD at 6 or 12 months of follow-up.

**Fig. 2:**
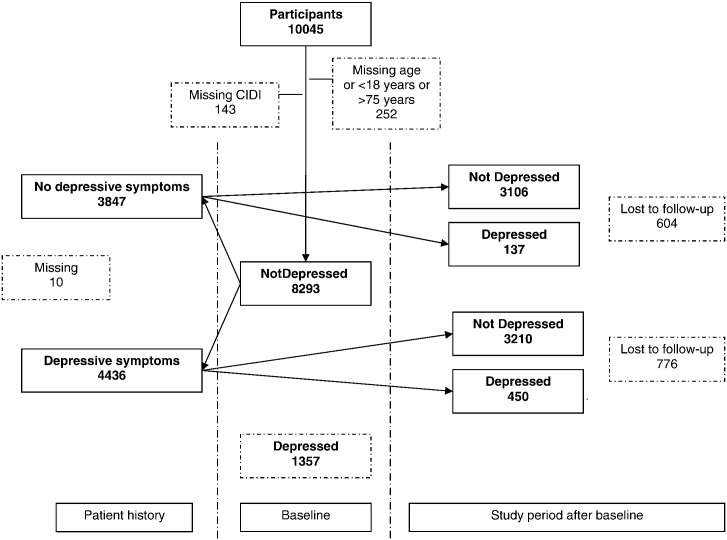
Flowchart of participants without major depressive disorder (MDD) in the 6 months prior to baseline and without a lifetime history of depressive symptoms, who have a possible *first* onset of MDD at 6 or 12 months of follow-up.

Fig. 1 shows that 6910 persons (83.3%) had a complete follow-up, of whom 589 (8.5%) had an onset of MDD at 6 or 12 months of follow-up. In the younger age group 166 persons (28%) had an onset of MDD at 6 or 12 months of follow-up, in the middle aged group 254 (43%) and in older age group 169 persons (29%) (Table 2). The risk of onset was the greatest in those aged 40 to 49 years. Attrition rates were similar for women (16.4%) and men (17.2%) and for those who were middle aged (16.0%) or older (15.7%) but those who were younger were slightly more likely to be lost to follow-up (18.2%). Other risk factors for onset of MDD at 6 or 12 months of follow-up were being female and having lower levels of education. Compared to the UK, persons from Spain and Chile were more likely to have an onset of MDD at 6 or 12 months of follow-up, while persons from Slovenia, Estonia and the Netherlands were less likely to have an onset of MDD at 6 or 12 months of follow-up. The risk of onset of MDD at 6 or 12 months of follow-up increased with increasing number of life events, and was increased for all types of life event. Fig. 2 shows that 137 out of 3243 persons (4.2%) with no MDD in the 6 months prior to baseline had a first onset of MDD at 6 or 12 months of follow-up in the absence of a lifetime history of MDD.

Table 3 shows the results of the logistic regression analyses for the independent and combined effects of age group (in tertiles) and life event on the risk of MDD, and the RERI with 95% CI. In the absence of a life event, persons in middle age (41 to 57 years) had the largest risk of MDD compared to those who were younger or older (e.g. OR 1.42; 95% CI 1.13 to 1.78 for no personal problems and middle age). If a life event was present, all types of life event showed the largest effect at age 41 to 57 years (e.g. OR 3.03; 95% CI 2.19 to 4.18 for personal problems and middle age). A statistically significant interaction between middle age and life event was found for personal problems (RERI = 1.30; 95% CI 0.29 to 2.32), severe events in family or friends (RERI = 1.23; 95% CI 0.28 to 2.19) and problems with law (RERI 1.57; 95% CI 0.33 to 2.82), indicating that the combined effect of middle age and life event on the risk of MDD was greater than the sum of the individual effects. No significant interaction between life events and older age was found for any of the life event categories. The results were similar when the models were adjusted for sex, level of education and country. In addition, a statistically significant interaction between two or more life events and middle age was found after adjustment for confounders. When we repeated the analyses in those with a possible first onset of MDD at 6 or 12 months of follow-up, all types of life event still showed the largest effect at middle age, although none of the RERIs was statistically significant (data available on request).

Table 4 shows the results of the logistic regression analyses for the independent and combined effects of the 10 year age groups and life event on the risk of MDD, and the RERI with 95% CI. The results are also presented in strata of sex and are adjusted for level of education and country. The most important finding is that all life event groups have the greatest effect at middle age, especially in women aged 40 to 49 years.

## 4 Discussion

In this large scale cross-national prospective cohort study in primary care attendees two main observations emerged: 1) life events, regardless type of life event, pose the largest risk on the onset of major depressive disorder in mid-life, especially in women aged 40 to 49 years; 2) the combined effect of personal problems, severe events in family or friends, or problems with law and middle age is larger than the sum of the individual effects.

Strengths of our study are that we used data from a prospective cohort study and thus were able to examine life events before onset of MDD. Also, our cohort was large and had a wide age range and included participants from 7 countries. We diagnosed MDD according to DSM-IV criteria using the same structured interview in all countries and identified life events from a widely used schedule. Furthermore, loss to follow-up was low in all age groups. Our study was limited by the lower response to recruitment in the UK and the Netherlands, which possibly occurred because the study was not as strongly endorsed by family doctors as in the other countries in the study (King et al., 2008b). Ethical constraints prevented the collection of data on non-responders at baseline. Although we excluded those who had dementia, we cannot rule out the possibility that cognitive impairment may have influenced the recalling of life events in those who were older. This may have led to an underestimation of life events in the oldest group and possibly to a weaker effect on the onset of MDD.

To our knowledge, only four studies have examined whether life events have a different effect in different age groups on the risk of depression. In one large epidemiological study among 3491 individuals aged 48 to 79 years, maternal loss had a greater effect in those who were middle aged compared to those who were older, which is comparable to our results (Surtees and Wainwright, 2000). Another large study among 8580 participants aged 16 to 74 years showed that recent threats to health, recent interpersonal problems and lifetime stressors were associated with common mental disorders (Jordanova et al., 2007). In particular, they found that the strength of association between recent life events and common mental disorders was the largest in those aged 45 to 54 years. Although the study had a cross-sectional design and included both depressive and anxious persons, the results were comparable to ours. Another study did not find an interaction between life events and age on the risk of onset of depression, but this study included only 64 patients with depression and 74 without depression and thus was likely to be limited in power (Oei and Zwart, 1990). The fourth study examined whether the impact of events (e.g. a recent divorce or suicide of a relative) on the risk of onset of depression varied with age in 13,006 patients who were admitted to a psychiatric ward for the first time (Kessing et al., 2003). Although this study did not find an interaction between more remote life events and age, a significant interaction was observed between age and a recent divorce that occurred within the last year before admission, and between age and being unmarried in the 2 year period before admission on the risk of a first depression.

Findings from the present study suggest that life events have the largest effect in mid-life on the risk of onset of MDD. The combined effect of all life event groups and 40 to 49 years of age had a statistically significant RERI, which means that the combined effects were larger than the sum of the individual effects. The interaction was statistically significant among women but not among men. Confidence intervals were wide in men, and the results in men should therefore be interpreted with caution. However, our results may suggest that those who are middle aged, and especially women, are more likely to become depressed if a recent personal problem occurs than younger or older people experiencing the same life event. These findings accord with the vulnerability-stress model of depression that states that stressors in combination with vulnerability levels are needed in order to be sufficient to cause depression (Holmes and Rahe, 1967; Krantz et al., 1985; Masuda and Holmes, 1967; Ormel, 1999). It could be that the middle aged is more vulnerable to the consequences of stressful life events (e.g. recent serious illness, assault or injury) than younger or older people (Jordanova et al., 2007). People in their middle age may have more responsibilities and social ties and thus life events at this stage of life could led to the onset of MDD even if they are more resilient than younger or older people as a consequence of their life experiences and in the absence of poor health and possible social isolation respectively. Moreover, women may have a greater biological vulnerability to the onset of MDD (Accortt et al., 2008) and sensitivity to hormonal changes of the menopause may further increase their vulnerability to onset of MDD compared to men (Dennerstein and Soares, 2008). Therefore, women in mid-life are the most susceptible to the onset of MDD, when a major negative life event occurs. Management of MDD in middle age people, and especially women, in clinical practice may require dealing with the effects of major life events on the treatment and prognosis of the disorder.

Although the effect of life events may be different for different age groups on the risk of onset of MDD, our findings showed that none of the RERIs was statistically significant for a *first* onset of MDD, although all types of life event showed the largest effect at middle age, independent of a history of depressive symptoms. It is possible that even larger prospective studies are needed to detect whether or not life events have a different effect in different age groups on the risk of a first depression. Future research should take account of the possibility that the effect of life events may be different on a first onset of depression compared to recurrent depression (Kessler, 2003).

In conclusion, the results from the present study suggest that recent life events carry the largest risk of onset of major depressive disorder in mid-life. Understanding the different vulnerability to life events according to age may help to indicate groups at a particular risk and assist in preventive strategies.

## Role of funding source

The study was funded by European Commission's Fifth Framework (Grant number PREDICT-QL4-CT2002-00683) and a VIDI grant from the Netherlands Organization for Scientific Research (NWO Project number 917-66-311). Partial support in Europe was the Slovenian Ministry for Research (grant 4369-1027), the Spanish Ministry of Health (Grant field-initiated studies program references PI041980, PI041771, and PI042450), the Spanish Network of Primary Care Research (redIAPP) (ISCIII-RETIC RD06/0018) and SAMSERAP group. The UK National Health Service Research and Development office provided service support costs in the United Kingdom. The funding sources had no further role in study design, in the collection, analysis and interpretation of data, in the writing of the report, and in the decision to submit the paper for publication.

## Conflict of interest

All authors report no conflicts of interest.

## Figures and Tables

**Table 1:** Characteristics for 8293 participants with no major depressive disorder in the 6 months prior to baseline.

	Total (N = 8293) %	Age 18 to 40 years (N = 2797) %	Age 41 to 57 years (N = 2780) %	Age 58 to 75 years (N = 2716) %
Socio-demographic				
Age in years, mean (SD)	49 (16)	30 (6)	49 (5)	66 (5)
Female	67	73	69	60
Education (lower)^a^	43	28	45	53
Country				
UK	14	10	13	18
Spain	12	10	12	15
Slovenia	13	11	15	12
Estonia	11	18	8	7
Netherlands	13	12	15	12
Portugal	12	11	12	14
Chile	26	29	25	23
Life events—number				
No	40	36	40	44
One	30	30	29	32
Two or more	30	34	31	25
Life events—groups				
Personal problems	15	12	15	17
Relational problems	39	38	39	39
Work/financial problems	14	19	14	9
Events in family/friends	21	29	23	11
Problems with law	8	10	8	7

All covariates had ≤ 1% missing data, except for severe events in family or friends (13%). Percentages may not add up to 100% due to rounding.

a Lower education was defined as primary school, trade or no education. Higher education (the reference category) was defined as secondary school or higher.

**Table 2:** Characteristics for those with and without onset of major depressive disorder at 6 or 12 months of follow-up.

	Total (N = 6910) %	No onset (N = 6321) %	Onset (N = 589) %	OR (95% CI)
Socio-demographic				
Age in years, tertiles				
18 to 40	33	34	28	1 (ref)
41 to 57	34	33	43	1.56 (1.27 to 1.92)⁎
58 to 75	33	34	29	1.02 (0.82 to 1.27)
18 to 29	15	15	11	1 (ref)
30 to 39	17	17	15	1.30 (0.93 to 1.81)
40 to 49	19	18	23	1.81 (1.33 to 2.47)⁎
50 to 59	21	21	25	1.69 (1.25 to 2.30)⁎
60 to 69	20	20	18	1.23 (0.89 to 1.70)
70 to 75	9	10	8	1.10 (0.74 to 1.64)
Female	68	66	80	2.00 (1.62 to 2.46)⁎
Education (lower)^a^	41	39	55	1.89 (1.59 to 2.24)⁎
Country				
UK	14	14	14	1 (ref)
Spain	10	10	18	1.83 (1.34 to 2.49)⁎
Slovenia	13	14	7	0.46 (0.31 to 0.68)⁎
Estonia	12	12	8	0.66 (0.46 to 0.96)⁎
Netherlands	14	14	8	0.57 (0.39 to 0.82)⁎
Portugal	12	12	12	0.95 (0.68 to 1.33)
Chile	25	24	33	1.35 (1.03 to 1.78)⁎
Life events—number				
No	41	42	28	1 (ref)
One	30	30	30	1.48 (1.18 to 1.84)⁎
Two or more	29	28	42	2.29 (1.87 to 2.82)⁎
Life events—groups^b^				
Personal problems	14	14	21	1.73 (1.40 to 2.13)⁎
Relational problems	38	37	46	1.46 (1.23 to 1.73)⁎
Work/financial problems	14	13	21	1.83 (1.48 to 2.26)⁎
Events in family/friends	20	19	31	1.90 (1.56 to 2.31)⁎
Problems with law	8	8	12	1.60 (1.22 to 2.09)⁎

OR = Odds ratio, CI = Confidence intervals. Percentages may not add up to 100% due to rounding.

a Lower education was defined as primary school, trade or no education. Higher education (the reference category) was defined as secondary school or higher.

b Absence of the life event for each group is the reference category (e.g. for the life event group of personal problems, no personal problems is the reference category).

⁎ P < 0.05.

**Table 3:** Logistic regression models with onset of major depressive disorder at 6 or 12 months of follow-up as dependent variable, and the life event groups, age (tertiles) and their interaction as independent variables.

	Onset of MDD at follow-up (N = 6910)		
	Odds ratio	RERI_crude_	RERI_adjusted_^a^
	(95% CI)	(95% CI)	(95% CI)
No life events and younger age	1 (Reference)		
No life events and middle age	1.59 (1.06 to 2.38)		
No life events and older age	1.25 (0.82 to 1.89)		
One life event and younger age	1.72 (1.12 to 2.63)		
One life event and middle age	2.42 (1.61 to 3.63)	0.11 (− 0.80 to 1.02)	0.06 (− 0.81 to 0.94)
One life event and older age	1.61 (1.05 to 2.47)	− 0.36 (− 1.21 to 0.50)	− 0.37 (− 1.20 to 0.45)
Two or more life events and younger age	2.28 (1.52 to 3.40)		
Two or more life events and middle age	4.03 (2.76 to 5.88)	1.16 (− 0.01 to 2.34)	0.96 (0.02 to 1.91)⁎
Two or more life events and older age	2.56 (1.68 to 3.90)	0.03 (− 0.94 to 1.01)	− 0.06 (− 0.94 to 0.82)
No personal problems and younger age	1 (Reference)		
No personal problems and middle age	1.42 (1.13 to 1.78)		
No personal problems and older age	0.97 (0.76 to 1.25)		
Personal problems and younger age	1.31 (0.83 to 2.06)		
Personal problems and middle age	3.03 (2.19 to 4.18)	1.30 (0.29 to 2.32)⁎	1.35 (0.31 to 2.40)⁎
Personal problems and older age	1.52 (1.05 to 2.22)	0.24 (− 0.54 to 1.02)	0.14 (− 0.61 to 0.89)
No relational problems and younger age	1 (Reference)		
No relational problems and middle age	1.66 (1.25 to 2.20)		
No relational problems and older age	1.18 (0.87 to 1.60)		
Relational problems and younger age	1.69 (1.23 to 2.32)		
Relational problems and middle age	2.43 (1.81 to 3.26)	0.09 (− 0.62 to 0.79)	0.08 (− 0.56 to 0.73)
Relational problems and older age	1.45 (1.04 to 2.01)	− 0.42 (− 1.07 to 0.24)	− 0.37 (− 0.96 to 0.22)
No work/financial problems and younger age	1 (Reference)		
No work/financial problems and middle age	1.61 (1.27 to 2.03)		
No work/financial problems and older age	1.08 (0.84 to 1.39)		
Work/financial problems and younger age	1.74 (1.22 to 2.50)		
Work/financial problems and middle age	2.98 (2.11 to 4.21)	0.63 (− 0.42 to 1.69)	0.65 (− 0.45 to 1.75)
Work/financial problems and older age	2.21 (1.39 to 3.50)	0.38 (− 0.71 to 1.48)	0.40 (− 0.74 to 1.54)
No events in family/friends and younger age	1 (Reference)		
No events in family/friends and middle age	1.46 (1.12 to 1.90)		
No events in family/friends and older age	1.16 (0.89 to 1.52)		
Events in family/friends and younger age	1.65 (1.18 to 2.31)		
Events in family/friends and middle age	3.34 (2.43 to 4.58)	1.23 (0.28 to 2.19)⁎	1.30 (0.35 to 2.25)⁎
Events in family/friends and older age	2.06 (1.30 to 3.26)	0.25 (− 0.74 to 1.24)	0.19 (− 0.73 to 1.12)
No problems with law and younger age	1 (Reference)		
No problems with law and middle age	1.46 (1.18 to 1.82)		
No problems with law and older age	0.99 (0.78 to 1.26)		
Problems with law and younger age	0.99 (0.57 to 1.71)		
Problems with law and middle age	3.02 (2.02 to 4.53)	1.57 (0.33 to 2.82)⁎	1.31 (0.10 to 2.51)⁎
Problems with law and older age	1.75 (1.04 to 2.95)	0.77 (− 0.25 to 1.79)	0.67 (− 0.31 to 1.65)

MDD = Major depressive disorder, OR = Odds ratio, CI = Confidence interval, RERI = Relative excess risk due to interaction. RERI formula = OR_A + B +_ − OR_A + B −_ − OR_A−B +_ + 1. RERI examples: Personal problems and middle age (3.03 − 1.31 − 1.42 + 1 = 1.30); personal problems and older age (1.52 − 1.31 − 0.97 + 1 = 0.24).

a Adjusted for sex, level of education and country.

⁎ Significant departure from additivity; note that in the absence of interaction as departure from additivity, RERI = 0.

**Table 4:** Logistic regression models with onset of major depressive disorder at 6 or 12 months of follow-up as dependent variable, and the life event groups, age in 10 year groups and their interaction as independent variables, stratified by sex.

	Onset of MDD at follow-up (N = 6910)		
	RERI_adjusted_ (95% CI)		
	Total	Women	Men
No life events and 18 to 29 years of age	(Reference)	(Reference)	(Reference)
One life event and 30 to 39 years of age	0.90 (0.08 to 1.72)⁎	1.05 (0.22 to 1.87)⁎	− 2.83 (− 12.38 to 6.72)
One life event and 40 to 49 years of age	0.89 (0.05 to 1.73)⁎	0.82 (0.02 to 1.62)⁎	0.34 (− 8.34 to 9.03)
One life event and 50 to 59 years of age	0.33 (− 0.67 to 1.32)	0.39 (− 0.56 to 1.34)	− 0.75 (− 9.11 to 7.61)
One life event and 60 to 69 years of age	0.48 (− 0.40 to 1.36)	0.32 (− 0.58 to 1.21)	1.39 (− 4.74 to 7.52)
One life event and 70 to 75 years of age	− 0.10 (− 1.13 to 0.93)	0.26 (− 0.73 to 1.24)	− 3.60 (− 14.10 to 6.89)
No life events and 18 to 29 years of age	(Reference)	(Reference)	(Reference)
Two or more life events and 30 to 39 years of age	0.71 (− 0.20 to 1.62)	0.74 (− 0.16 to 1.63)	− 0.42 (− 14.33 to 13.49)
Two or more life events and 40 to 49 years of age	2.63 (1.22 to 4.03)⁎	2.29 (1.02 to 3.55)⁎	10.14 (− 12.02 to 32.30)
Two or more life events and 50 to 59 years of age	0.13 (− 0.81 to 1.06)	0.33 (− 0.58 to 1.26)	− 2.08 (− 10.48 to 6.33)
Two or more life events and 60 to 69 years of age	0.55 (− 0.63 to 1.72)	0.67 (− 0.20 to 1.54)	0.39 (− 7.20 to 6.42)
Two or more life events and 70 to 75 years of age	0.53 (− 0.68 to 1.74)	0.40 (− 0.80 to 1.60)	2.03 (− 6.97 to 11.03)
No personal problems and 18 to 29 years of age	(Reference)	(Reference)	(Reference)
Personal problems and 30 to 39 years of age	0.10 (− 1.28 to 1.49)	0.25 (− 1.13 to 1.62)	− 0.34 (− 6.67 to 5.99)
Personal problems and 40 to 49 years of age	2.23 (0.33 to 4.13)⁎	2.66 (0.55 to 4.76)⁎	− 1.78 (− 9.83 to 6.28)
Personal problems and 50 to 59 years of age	0.86 (− 0.47 to 2.20)	0.61 (− 0.75 to 1.99)	1.86 (− 4.33 to 8.05)
Personal problems and 60 to 69 years of age	0.78 (− 0.45 to 2.04)	0.82 (− 0.52 to 2.15)	0.31 (− 5.07 to 5.68)
Personal problems and 70 to 75 years of age	− 0.63 (− 1.98 to 0.72)	− 0.48 (− 1.82 to 0.85)	− 1.99 (− 7.88 to 3.90)
No relational problems and 18 to 29 years of age	(Reference)	(Reference)	(Reference)
Relational problems and 30 to 39 years of age	0.39 (− 0.35 to 1.13)	0.44 (− 0.32 to 1.21)	− 0.10 (− 2.78 to 2.57)
Relational problems and 40 to 49 years of age	1.12 (0.23 to 2.01)⁎	1.00 (0.16 to 1.85)⁎	2.17 (− 2.76 to 7.08)
Relational problems and 50 to 59 years of age	− 0.29 (− 1.16 to 0.58)	− 0.09 (− 0.97 to 0.79)	− 0.89 (− 4.23 to 2.45)
Relational problems and 60 to 69 years of age	− 0.03 (− 0.83 to 0.76)	− 0.02 (− 0.83 to 0.78)	− 0.01 (− 3.09 to 3.07)
Relational problems and 70 to 75 years of age	− 0.03 (− 0.94 to 0.87)	− 0.08 (− 1.05 to 0.89)	0.20 (− 2.54 to 2.95)
No work/financial problems and 18 to 29 years of age	(Reference)	(Reference)	(Reference)
Work/financial problems and 30 to 39 years of age	0.41 (− 0.96 to 1.78)	0.40 (− 1.07 to 1.86)	0.16 (− 3.78 to 4.11)
Work/financial problems and 40 to 49 years of age	1.85 (0.07 to 3.63)⁎	1.47 (− 0.24 to 3.18)	4.82 (− 4.70 to 14.35)
Work/financial problems and 50 to 59 years of age	0.20 (− 1.19 to 1.60)	0.45 (− 1.20 to 2.09)	0.75 (− 3.50 to 4.99)
Work/financial problems and 60 to 69 years of age	0.57 (− 0.99 to 2.14)	1.21 (− 0.63 to 3.05)	− 3.42 (− 8.14 to 1.29)
Work/financial problems and 70 to 75 years of age	1.36 (− 1.62 to 4.35)	1.39 (− 2.30 to 5.07)	1.98 (− 4.67 to 8.64)
No events in family/friends and 18 to 29 years of age	(Reference)	(Reference)	(Reference)
Events in family/friends and 30 to 39 years of age	0.39 (− 0.63 to 1.41)	0.46 (− 0.56 to 1.49)	− 1.33 to (− 7.87 to 5.21)
Events in family/friends and 40 to 49 years of age	2.66 (1.08 to 4.23)⁎	2.59 (1.03 to 4.15)⁎	2.29 (− 5.43 to 10.00)
Events in family/friends and 50 to 59 years of age	0.40 (− 0.64 to 1.44)	0.95 (− 0.25 to 2.16)	− 1.90 (− 7.76 to 3.95)
Events in family/friends and 60 to 69 years of age	0.30 (− 0.90 to 1.50)	0.38 (− 0.83 to 1.59)	− 0.35 (− 6.11 to 5.41)
Events in family/friends and 70 to 75 years of age	1.51 (− 0.92 to 3.93)	1.52 (− 1.10 to 4.15)	1.05 (− 7.25 to 9.35)
No problems with law and 18 to 29 years of age	(Reference)	(Reference)	(Reference)
Problems with law and 30 to 39 years of age	0.20 (− 1.12 to 1.52)	0.33 (− 0.98 to 1.64)	− 1.24 (− 6.62 to 4.13)
Problems with law and 40 to 49 years of age	3.87 (1.21 to 6.53)⁎	3.33 (0.76 to 5.91)⁎	8.52 (− 5.56 to 22.60)
Problems with law and 50 to 59 years of age	− 0.08 (− 1.21 to 1.05)	0.15 (− 1.14 to 1.45)	− 1.64 (− 7.14 to 3.87)
Problems with law and 60 to 69 years of age	1.12 (− 0.34 to 2.58)	1.67 (− 0.08 to 3.41)	− 1.55 (− 6.68 to 3.57)
Problems with law and 70 to 75 years of age	1.07 (− 1.00 to 3.14)	1.34 (− 1.04 to 3.71)	− 0.06 (− 5.92 to 5.80)

MDD = major depressive disorder, CI = confidence interval, RERI = relative excess risk due to interaction. RERI formula = OR_A+B +_ − OR_A + B −_ − OR_A−B +_ + 1. All models are adjusted for level of education and country.

⁎ Significant departure from additivity; note that in the absence of interaction as departure from additivity, RERI = 0.
